# Encephalitis in the Course of HHV-7 Infection in an Infant

**DOI:** 10.3390/jcm13020418

**Published:** 2024-01-12

**Authors:** Justyna Moppert, Eliza Łężyk-Ciemniak, Małgorzata Pawłowska

**Affiliations:** 1 Department of Infectious Diseases and Hepatology, Collegium Medicum, Bydgoszcz, Nicolaus Copernicus University, 87-100 Torun, Poland; mpawlowska@cm.umk.pl; 2 Department of Paediatrics, Infectious Diseases and Hepatology, Voivodeship Infectious Observation Hospital, 85-030 Bydgoszcz, Poland; elizalezyk@wp.pl

**Keywords:** HHV-7, encephalitis, children

## Abstract

Most cases of acute infections caused by human herpesvirus 7 (HHV-7) are asymptomatic or very mild. Clinical symptoms disappear spontaneously; however, the infection becomes latent and persists for life with periodic asymptomatic reactivation. Little is known about the virus’s ability to cross the blood–brain barrier. Our case of an immunocompetent infant indicates that HHV-7 infection should be considered a cause of neuroinfection, not only in immunocompromised patients but also in the youngest immunocompetent patients.

## 1. Introduction

Human herpesvirus 7 (HHV-7), a virus belonging to the *Herpesviridae* family, was first isolated in 1990 from the T lymphocytes of a healthy blood donor. Serological exponents of past HHV-7 infection are found in approximately 98% of adults. Primary HHV-7 infection usually occurs between two and five years of age. Approximately 65% of children up to the age of two and about 90% of children up to the age of five are seropositive [1]. HHV-7, like other viruses of the *Herpesviridae* family, causes primary infection, either symptomatic or asymptomatic, which then progresses to latent infection. The virus replicates and induces a cytopathic effect, primarily in CD4 and CD8 T lymphocytes [2]. The course of the infection is generally asymptomatic. The most common clinical manifestation is a three-day fever known as exanthem subitem. Sources of infection are usually asymptomatic carriers. HHV-7 infections occur mainly by the droplet and sexual route and less commonly indirectly through objects freshly contaminated with secretions and excretions. There is no evidence that HHV-7 causes congenital infections, but it is present in the milk of 10% of lactating women. Severe HHV-7-induced morbidity occurs most often as a result of a so-called ‘delayed primary infection’ in older children and adults with cell-mediated immune disorders. There are few data to elucidate the mechanism by which HHV-7 crosses the blood–brain barrier and the pathomechanism of the neuroinfection it causes [2,3]. The clinical picture of central nervous system (CNS) involvement is not pathognomonic. The range of clinical signs of CNS involvement includes febrile convulsions, encephalitis, meningitis, facial nerve palsy, vestibular nerve inflammation, qualitative and quantitative disturbances of consciousness, ataxia, and coma.

## 2. Detailed Case Description

An 11-month-old immunocompetent boy was admitted to the Department of Paediatrics, Infectious Diseases, and Hepatology due to persistent fever, irritability, vomiting, and diarrhoea with blood admixture for two days. The boy was vaccinated according to the immunisation calendar, and the perinatal and family histories were unremarkable. At three months of age, he contracted COVID-19. The clinical manifestations of severe acute respiratory-associated coronavirus-2 (SARS-CoV-2) infection were fever and diarrhoea. On admission, the child showed dehydration and increased lethargy. There were no meningeal symptoms or features of focal central or peripheral nervous system damage. Baseline laboratory tests on the day of hospitalisation showed leucocytosis with granulocytosis, hypoalbuminaemia, and slightly elevated C-reactive protein (CRP) levels (Table 1).

Virological and microbiological stool tests for rota-, adeno-, and norovirus and Campylobacter (determined by qualitative cassette immunochromatographic test, BioMaxima) were negative. Stool cultures for Salmonella, Shigella, and Yersinia infections were negative. The patient’s serum showed antibodies against COVID-19 in the IgG class. No significant pathologies were described in the abdominal ultrasound or chest X-ray. On the first day of hospitalisation, a lumbar puncture was performed, yielding lymphocytic cerebrospinal fluid (CSF) (Table 2).

Neuroborreliosis was excluded in the differential diagnosis on the basis of the absence of specific IgM/IgG antibodies to Borrelia burgdorferi sensu lato in the CSF (determined by indirect chemiluminescence CLIA, DiaSorin LIAISON^®^, Saluggia, Italy). Therapy included empirical acyclovir (20 mg/kg/dose every 8 h) and dexamethasone (0.15 mg/kg/dose every 6 h) from the first day. Real-time PCR (RT-PCR) in the CSF allowed for the exclusion of enterovirus, parechovirus, herpes simplex virus (HSV) 1/2, varicella zoster virus (VZV), parvovirus B19, Epstein–Barr virus (EBV), cytomegalovirus (CMV), and human herpesvirus 6 (HHV-6) infection. The presence of the genetic material of HHV-7 was confirmed. To determine EBV, CMV, HSV1/2, VZV, parvovirus B19, ADV, and HHV-6 viral load TaqMan Fast Advanced Master Mix probes were used; to determine enterovirus, parechovirus and HHV-7 viral load TaqPath 1-Step RT-qPCR Master Mix probes were used, according to the manufacturer’s protocol (ThermoFisher, Waltham, MA, USA). As specific IgM class antibodies against HHV-7 are not detected in primary infection in children, although IgG class antibodies appeared from the second week of clinical symptoms in the analysed patient, serological tests were not performed. The diagnosis of neuroinfection with HHV-7 aetiology was based on the presence of HHV-7 genetic material in the CSF. After confirmation of HHV-7 aetiology, ganciclovir (16 mg/kg/dose) was administered on the third day of hospitalisation, and systemic steroids were continued.

Numerous diffuse foci of demyelination in the parietal, temporal, and occipital lobes and in the hippocampus were found in the magnetic resonance imaging (MRI) of the head, which was performed on the third day of ganciclovir therapy. Due to the changes found in the MRI of the head, human immunoglobulins (Privigen) (0.4 g/kg/dose for five days) were used from the sixth day of the ganciclovir treatment. A follow-up MRI of the head performed on day 26 of the ganciclovir treatment showed a significant reduction in demyelination foci. Antiviral treatment was continued for six weeks based on an assessment of the drug’s pharmacokinetics. A post-therapy neurological follow-up examination revealed delayed psychomotor development in the child. After 45 days of hospitalisation, the patient was discharged home with the recommendation of further rehabilitation in an outpatient setting.

## 3. Discussion

The clinical picture of meningitis and encephalitis is heterogeneous and depends on the patient’s age. The classic symptoms including high fever, headache, nausea, vomiting, irritability, hyperesthesia, and photophobia, typically found in older children and adults, are often absent in the youngest patients. In the first year of life, the disease may manifest itself with fever or hypothermia (poor prognostic factor), jaundice, decreased appetite, vomiting, diarrhoea, convulsions, irritability, or apathy. In the described case, vomiting and diarrhoea were the main causes of hospitalization.

The association of HHV-7 infection with the development of encephalitis in both immunocompetent and immunocompromised patients has been demonstrated for delayed primary HHV-7 infection [1,2]. Schwartz et al., based on an analysis of the records of 2972 paediatric patients hospitalised for neurological disorders between 1998 and 2011, demonstrated the presence of HHV-7 DNA genetic material in the CSF of 57 (1.9%) children. The mean age of the patients was 10 years, and no gender predominance was found. All children in this group presented with symptoms of meningitis. The analysis was divided into probable and definite HHV-7 infections. Probable infections were considered cases in which patients presented clinical symptoms and HHV-7 genetic material was found in the CSF, but the serum was not analysed for specific antibodies. Serological exponents of primary HHV-7 infection found in the serum (presence of IgG antibodies with low avidity, and IgM antibodies were not assessed) were the basis for a confident diagnosis, which included three immunocompetent children. Two were diagnosed with encephalitis, and one was diagnosed with Guillain–Barre syndrome (GBS). This is the first report linking primary HHV-7 infection to the development of GBS [1]. 

Foiadelli et al. also demonstrated an association between HHV-7 infection and CNS damage [2]. In a group of 12 patients with acute encephalopathy, the presence of HHV-7-DNA genetic material was confirmed in the cerebrospinal fluid using RT-PCR. Seven children showed symptoms of meningitis and encephalitis. In this group, acute demyelinating encephalitis (ADEM) was confirmed in two patients, and acute haemorrhagic encephalitis (AHEM) was confirmed in one patient. The remaining five patients showed acute neuropsychiatric symptoms in the form of paediatric acute-onset neuropsychiatric syndrome (PANS). The mean age of the children in Foiadelli’s study was 9.5 years, which was similar to the mean age of the children analysed by Schwartz. In the Schwartz study, the presence of low-avidity IgG antibodies was required in addition to an assessment of the viral load in the CSF to make a confident diagnosis. In the Foiadelli study, the basis for diagnosis was the assessment of the HHV-7 viral load in the CSF and serum, which were not assessed for serological exponents. Indeed, the interpretation of serological tests is complicated by the high level of cross-reactivity between viruses of the *Herpesviridae* family. In the presented clinical case, no serological tests were performed. The diagnosis was based on confirmation of the presence of HHV-7 genetic material in the CSF. Detection of HHV-7 in CSF does not necessarily indicate a free virus. RT-PCR will detect genetic material of the virus, whether it is intracellular or extracellular. Therefore, the lack of assessment of the increase in the titre and avidity of IgG antibodies, which is helpful in the differential diagnosis of primary active infection and the reactivation of primary infection, is a limitation of this study. 

Parra et al. presented the importance of the molecular method in the diagnosis of neuroinfection with HHV-7 aetiology. They analysed the case of a 32-year-old immunocompetent man who was diagnosed with encephalitis with HHV-7 aetiology. The diagnosis was based on the evaluation of the HHV-7 viral load in the CSF. In a retrospective analysis, IgG class antibody titres from days 3, 14, and 21 were compared. An increase in titres from 1:32 to 1:64 was observed [4]. Therefore, the retrospective assessment of IgG antibody titres allowed differentiation of whether neuroinfection was the result of primary infection (including delayed infection) or a consequence of reactivation. However, in adult patients, 98% of whom have serological exponents of past HHV-7 infection, this method should not be used as a basis for diagnosis. Molecular methods allow rapid diagnosis and eliminate the possibility of cross-reactions and false positives. This is important, especially in the youngest patients, because although serological methods can be used to detect primary HHV-7 infection, immunological cross-reactivity between HHV-6 and HHV-7 may contribute to a high number of false-positive results due to the high proportion of children with HHV-6 infection (almost 100% by the age of three years) [5].

Schwartz, Foiadelli, and Ward noted that delayed primary infection (in children over five years of age) is associated with a higher incidence of severe neurological complications [1,2,6]. The reason delayed primary HHV-7 infection is characterised by a more severe course is the more aggressive inflammatory response produced by a mature immune system, as is the case with other virus-induced diseases (e.g., chickenpox, rubella, and measles). The development of neurological complications during HHV-7 infection is not due to the direct viral action within the CNS but is rather the result of an abnormal immune response and may be related to TCD8+ lymphocyte-dependant cytotoxicity [2]. During viral infections, the cells of the acquired immune response become activated when they recognise antigens through their antigen-specific receptors. The T cell receptor (TCR) recognises antigens as small peptides bound to major histocompatibility complex (MHC) molecules on the surface of antigen-presenting cells (APCs). An important component of innate immunity that initiates the immune response, including autoimmunity, is the activation of Toll-like receptors (TLRs) on APCs via the breakdown products of pathogens. More than a dozen TLRs are known, some of which play an important role in the pathogenesis of autoimmune diseases. These receptors, when combined with appropriate ligands, activate the immune response through the induction and synthesis of pro-inflammatory cytokines and the increased expression of major tissue compatibility system antigens and adhesion molecules. Dendritic cells maturing under TLR stimulation become resistant to the suppressive effects of regulatory T cells, which may result in a breakdown of tolerance to autoantigens. TLRs, through their influence on Th1, Th2, and Th17 helper lymphocyte subpopulations, play an important role in controlling acquired immunity. CNS damage in the course of delayed primary HHV-7 infection may therefore resemble the pathomechanism of multiple sclerosis (MS) development. Patients with acute-onset MS have autoreactive T lymphocytes that produce proinflammatory cytokines. Their activation occurs peripherally and may result from cross-reactivity during, for example, a viral infection (molecular mimicry). According to this theory, infection with a virus or bacterium with epitopes showing similarity to host antigens can lead to the activation of autoreactive lymphocytes [7]. The history of a SARS-CoV-2 infection at four months of age (molecularly confirmed infection, with the presence of IgG COVID-19 antibodies confirmed during hospitalisation) may also have influenced the course of the infection in the patient described. COVID-19 infection results in the decreased activity of CD4+ T lymphocytes, CD8+ T lymphocytes, B lymphocytes, and natural killer (NK) cells. T cell dysfunction during SARS-CoV-2 infection reduces the ability to suppress viral expression and replication due to an impaired ability to maintain the latency phase. Therefore, a previous COVID-19 infection may not only increase the risk of reactivation of the primary infection with a virus from the *Herpesviridae* family but may also influence the course of the primary infection. This would occur by limiting the ability to achieve latency, thereby increasing the possibility of replication and expansion of the virus during primary infection [8].

The interference of SARS-CoV-2 in the dysregulation of the immune system may be one of the factors involved in the reactivation of primary latency-capable virus infections [9,10]. Therefore, the immaturity of the immune system related to the patient’s age and history of SARS-CoV-2 infection, further affecting CD4/CD8 lymphocyte suppression, may have had a significant impact on the development of neurological complications in the case studied. 

In 2022, Ikeda-Murakami et al. analysed nine cases of HHV-7 encephalitis available in the international literature [11]. In this group, only three patients were children, aged 6–12 years. To date, HHV-7 encephalitis in infants has not been described. Our paper presents the first such case in an 11-month-old immunocompetent child. 

## 4. Conclusions

It seems reasonable to consider HHV-7 as an aetiological factor in the differential diagnosis of neuroinfection, even in the youngest group of children. In this group of patients, a history of SARS-CoV-2 infection may alter the immune system response to HHV-7 infection, thereby creating the possibility of severe neurological complications.

## Figures and Tables

**Table 1:** Laboratory tests performed on the patient during hospitalisation.

Parameters	References Values	Day of Hospitalisation					
		24.06	27.06	02.07	07.07	26.07	05.08
WBC [10^3^/µL]	4.50–13.00	17.94	11.33	8.26	7.59	7.97	4.60
Granulocytes [10^3^/µL]	1.50–7.50	10.03	4.11	3.03	3.04	1.04	0.83
Lymphocytes [10^3^/µL]	1.50–8.00	6.33	5.00	3.88	3.96	5.90	3.29
Hgb [g/dL]	11.0–14.0	10.8	9.3	9.9	10.7	10.8	10.4
Platelets [10^3^/µL]	130–370	397	554	434	703	393	343
CRP [mg/L]	0.0–5.0	9.9	1.1	-	<0.06	-	-
Creatinine [mg/dL]	0.17–0.42	0.27	0.48	-	0.27	<0.20	0.24
ALT [U/L]	0–57	11	14	-	15	15	43
INR	0.95–1.2	1.09					
Albumins [g/dL]	4.02–4.76	2.73				3.76	

WBC: white blood cells; CRP: C-reactive protein; ALT: alanine transaminase; INR: international normalised ratio blood test.

**Table 2:** General examination of cerebrospinal fluid (CSF).

Parameters	CSF Value	Reference Values
Colour	Colourless/clear	Colourless/clear
Protein [mg/dL]	27	20–40
Glucose [mg/dL]	75	45–80
Lactates [mg/dL]	13	10–25
Chlorides [mmol/L]	125	113–127
Cytosis [/µL]	42	<=5
Granulocytes [%]	3	-
Lymphocytes [%]	97	-

## Data Availability

Data supporting the reported results can be provided upon request from the corresponding author.
